# Adrenal Crisis Induced by Zoledronic Acid in Two Patients With Hypopituitarism: A Case Report and Literature Review

**DOI:** 10.1155/crie/8854575

**Published:** 2026-01-08

**Authors:** Abrar Mohammed AlFaifi, Wael Mohammad AlMistehi

**Affiliations:** ^1^ Obesity Endocrine Metabolic Center, King Fahad Medical City, Riyadh, Saudi Arabia, kfmc.med.sa

**Keywords:** adrenal crisis, adrenal insufficiency, bisphosphonates, case series, zoledronic acid

## Abstract

Adrenal insufficiency (AI) is characterized by inadequate steroid hormone production and is frequently a consequence of hypopituitarism, which is also associated with increased risk of osteoporosis due to deficiencies in growth hormone, gonadotropins, and other pituitary hormones. Zoledronic acid (ZA), a widely used bisphosphonate, is associated with acute phase reaction (APR) that may trigger adrenal crisis in susceptible individuals. We describe two patients with hypopituitarism and osteoporosis who developed adrenal crisis shortly after their first ZA infusion, despite stable physiological steroid replacement and acetaminophen prophylaxis. One presented with hypotension and shock within 24 h, the other with hypotension, severe hyponatremia, and seizures at 48 h. Both recovered after high‐dose glucocorticoids and were later switched to denosumab without complications. These cases highlight the potential for adrenal crisis in patients with central AI receiving ZA and suggest that standard prophylaxis may be insufficient. Alternative therapies and enhanced precautions may be warranted in this vulnerable population.

## 1. Introduction

Adrenal insufficiency (AI) is characterized by the inadequate production of steroid hormones by the adrenal glands. Patients can have primary AI due to a defect in the adrenal glands or secondary AI resulting from a defect in the pituitary gland [1]. Several conditions can lead to secondary AI. Examples include the use of exogenous steroids for other chronic conditions, Sheehan’s syndrome, and pituitary macroadenomas, which may necessitate surgical intervention or radiation therapy. These treatments can impair pituitary function and potentially result in hypopituitarism. Drug‐induced secondary AI is another important consideration, particularly in elderly patients with polypharmacy. Thyroxine therapy has been reported to precipitate adrenal crisis in individuals with unrecognized pituitary or adrenal dysfunction [2]. In addition, medications that induce CYP3A4 activity, including rifampicin, carbamazepine, and phenytoin, may accelerate cortisol metabolism and contribute to AI [3].

Patients with hypopituitarism have an increased risk of developing osteoporosis. This is due to the deficiency of pituitary hormones and chronic use of replacement therapy [4]. Registry data show that adults with growth hormone deficiency have a markedly higher fracture risk than controls [5], and a nationwide study demonstrated increased osteoporotic, vertebral, and hip fractures in older patients with panhypopituitarism [6]. These patients are considered candidates for bisphosphonate treatment. Zoledronic acid (ZA), which is generally well tolerated, is associated with an acute phase reaction (APR) characterized by fever, headaches, and myalgia [7]. ~33% of patients experience an adverse reaction after their first dose, but this rate decreases with each additional infusion [8].

An APR may trigger an adrenal crisis in patients with underlying AI; however, evidence on this subject remains scarce. To date, only three cases have been reported: two patients with primary and one with secondary AI [9–11]. Our study adds two further cases, making it the first to specifically highlight this risk in individuals with hypopituitarism despite stable glucocorticoid replacement.

### 1.1. Case 1

A 60‐year‐old woman with Sheehan’s syndrome was diagnosed at the age of 29 at another institution, following a complicated delivery with severe postpartum hemorrhage. She subsequently developed failure to lactate, amenorrhea, fatigue, and weight loss and was diagnosed with panhypopituitarism (growth hormone deficiency, hypogonadotropic hypogonadism, central AI, and central hypothyroidism). An MRI of the pituitary demonstrated an empty sella, and she has remained clinically stable on lifelong hormone replacement over the past three decades. Her home medications included hydrocortisone 10 mg daily (body surface area = 1.67 m^2^), levothyroxine 75 µg daily, and vitamin D and calcium supplements, and she was counseled on doubling the steroid dose during an acute illness. She was also taking 20 mg of rosuvastatin daily for dyslipidemia. She developed severe osteoporosis (T‐score of −3.9 at the lumbar spine and −2.6 at the femoral neck) but without fragility fractures, and ZA was initiated.

On the day of the infusion, she was in her usual state of health. The patient’s vital signs were stable. Her physical examination and baseline laboratory results, including kidney function, thyroid function, calcium, and vitamin D were unremarkable (Table 1). She received an intravenous dose of 5 mg/100 mL of ZA along with normal saline and intravenous paracetamol according to our protocol. The following day, she presented to the emergency department with nausea, vomiting, and diarrhea. She rapidly deteriorated and became lethargic and nonresponsive to verbal stimuli. Her vital signs revealed hypotension (77/58 mmHg), fever (102.6°F), tachypnea (30 breaths per minute), and tachycardia (102 beats per minute). She was immediately resuscitated with fluid replacement and started on intravenous hydrocortisone 200 mg, followed by 100 mg thrice daily. Despite this, her blood pressure remained low, necessitating phenylephrine infusion. She was kept on empirical antibiotics since sepsis was a possible culprit but was later excluded. She had lactic acidosis and mild transaminitis; however, her sodium, potassium, and glucose levels were normal.

**Table 1:** Laboratory profile of Case 1.

Laboratory parameters	Baseline	Day 1	Day 2	Day 16	Reference range
Hemoglobin (g/dL)	13.6	12.7	—	13	12.3–15.3
Red blood cells (K/mm^3^)	4.71	4	4.6	4.7	4.10–5.10
Hematocrit (%)	41	40	41	42.3	35.9–44.6
Platelets (K/mm^3^)	263	320	320	230	150–450
White blood cells (K/mm^3^)	6.87	8.4	8.1	7.1	4.0–11.0
Neutrophils absolute (K/mm^3^)	2.84	3.4	3.2	2.7	1.8–7.8
Lymphocytes absolute (K/mm^3^)	3.36	3.4	3.7	3.2	1.0–4.8
Monocytes absolute (K/mm^3^)	0.51	0.53	0.51	0.4	0.0–0.8
Eosinophils absolute (K/mm^3^)	0.2	0.3	0.3	0.3	0.0–0.5
Basophils absolute (K/mm^3^)	0.07	0.1	0.09	0.04	0.0–0.2
Sodium (mmol/L)	139	134	137	141	136–145
Potassium (mmol/L)	4.21	4	4	3.8	3.5–5.2
Chloride (mmol/L)	105	110	109	102	101–111
Carbon dioxide (mmol/L)	24	16.90	11.80	23	22–29
Corrected calcium (mmol/L)	2.35	2.23	2.2	2.4	2.1–2.55
Magnesium (mmol/dL)	0.92	0.8	0.8	0.91	1.6–2.4
Blood urea nitrogen (mg/dL)	5.20	6	5.6	5	3.5–7.2
Creatinine (µmol/L)	64	120	88	67	40–90
Glomerular filtration rate (mL/min/1.73 m^2^)	80	66	74	84	>60
Glucose (mg/dL)	88.2	106	97	110	82–115
Alkaline phosphatase (U/L)	48	49	54	45	40–150
Alanine aminotransferase (U/L)	15	60	63	32	0.00–55
Aspartate aminotransferase (U/L)	19	42	42	21	5–34
Total bilirubin (µmol/L)	14	26.20	20.5	13	3.40–20.50
Direct bilirubin (µmol/L)	4	13.3	13	5.7	0.00–8.60
Lactate (mmol/L)	0.81	3.655	1.7	—	0.5–2.0
Troponin I (ng/L)	3	4.9	—	—	≤15
Thyroid stimulating hormone (m[iU]/L)	<0.0100	<0.0100	—	<0.0100	0.40–4.50
Free T4 (pmol/L)	13.4	15.10	—	15.7	9–19
Vitamin D (nmol/L)	88	95	—	97	>50
Leukocytes, urine	—	0	—	—	—
Nitrates, urine	—	0	—	—	—
Urine culture	—	Negative	—	—	—
Blood culture	—	Negative	—	—	—

The next day, her condition stabilized, and she was administered oral hydrocortisone (10 mg twice daily). 1 year after the event, she was switched to denosumab injections administered every 6 months, and to date, she has received three doses without any complications.

### 1.2. Case 2

A 52‐year‐old female had a giant prolactinoma. She had previously undergone transsphenoidal surgery and external beam radiation therapy, which were complicated by empty sella and hypopituitarism (growth hormone deficiency, central hypothyroidism, and central adrenal insufficiency). Her daily medications were hydrocortisone 10 mg in the morning and 5 mg in the evening (body surface area = 1.61 m^2^), levothyroxine 100 µg, and vitamin D and calcium supplements. She was also on atorvastatin 10 mg daily for dyslipidemia and perindopril 10 mg and amlodipine 5 mg daily for hypertension, and she was counseled on doubling the steroid dose during an acute illness. She was found to have osteoporosis (T‐score −2.7 at the lumbar spine and −1.8 at the femoral necks) and was scheduled for ZA therapy.

She received the initial dose in a manner similar to that of the first patient. Her vital signs were stable, except for elevated blood pressure (165/79 mmHg). Her baseline laboratory results, including kidney function, thyroid function, calcium, and vitamin D were unremarkable (Table 2). The first day after the infusion, the patient started experiencing flu‐like symptoms. On day 2, she experienced a sudden onset of lethargy and loss of consciousness. Her husband discovered that she was seizing and brought her to the emergency department. On arrival, she was unconscious, agitated, and hypotensive (88/35 mmHg). Her laboratory results revealed severe hyponatremia (112 mmol/L), which responded to the administration of 3% hypertonic saline and improved to 124 mmol/L. She was also administered intravenous hydrocortisone (100 mg), followed by 50 mg thrice daily.

**Table 2:** Laboratory profile of Case 2.

Laboratory parameters	Baseline	Day 2	Day 20	Reference range
Hemoglobin (g/dL)	11.6	12.7	12.3	12.3–15.3
Red blood cells (K/mm^3^)	4.5	5.01	4.89	4.10–5.10
Hematocrit (%)	39	36	35	35.9–44.6
Platelets (K/mm^3^)	251	214	207	150–450
White blood cells (K/mm^3^)	5.31	3.5	3.6	4.0–11.0
Neutrophils absolute (K/mm^3^)	2.3	1.15	1.69	1.8–7.8
Lymphocytes absolute (K/mm^3^)	3.38	1.8	1.28	1.0–4.8
Monocytes absolute (K/mm^3^)	0.37	0.6	0.41	0.0–0.8
Eosinophils absolute (K/mm^3^)	0.35	0.15	0.13	0.0–0.5
Basophils absolute (K/mm^3^)	0.07	0.05	0.03	0.0–0.2
Sodium (mmol/L)	138	112	134	136–145
Potassium (mmol/L)	3.7	3.9	4.2	3.40–4.40
Chloride (mmol/L)	105	96	104	98–107
Carbon dioxide (mmol/L)	23	30	24	22–29
Corrected calcium (mmol/L)	2.26	2.23	2.4	2.1–2.55
Magnesium (mmol/L)	—	0.65	0.88	0.66–1.07
Blood urea nitrogen (mmol/L)	4	4.6	4	3.5–7.2
Creatinine (µmol/L)	56	47	43	49–90
Glomerular filtration rate (mL/min/1.73 m^2^)	103	112	117	>60
Glucose (mg/dL)	81	71	100	82–115
Alkaline phosphatase (U/L)	84	93	—	40–150
Alanine aminotransferase (U/L)	10	39	24	0.00–55.00
Aspartate aminotransferase (U/L)	15	27	19	5–34
Total bilirubin (µmol/L)	4.4	7.4	—	3.40–20.50
Direct bilirubin (µmol/L)	2.8	4	—	0.0–8.6
Lactate (mmol/L)	—	4.2	0.8	0.5–2.2
Troponin I (ng/L)	—	2	2.3	≤15.60
Thyroid stimulating hormone (m[iU]/L)	0.012	<0.01	<0.01	0.40–4.50
Free T4 (pmol/L)	20	18	16	9–19
Vitamin D (nmol/L)	105	—	—	>50
Leukocytes, urine	—	0	—	—
Nitrates, urine	—	0	—	—
Urine culture	—	Negative	—	—
Blood culture	—	Negative	—	—

Her level of consciousness improved following resuscitation and intravenous hydrocortisone, with blood pressure stabilizing and sodium levels returning to normal. She was treated with oral hydrocortisone and discharged with her regular home dose. She later visited our clinic and was in good health with normal electrolyte levels. When she was due for her next antiresorptive therapy, she received denosumab and tolerated it well without complications.

## 2. Discussion

ZA is a bisphosphonate that reduces bone resorption by blocking the farnesyl pyrophosphate synthase pathway in osteoclasts, leading to the accumulation of isopentenyl pyrophosphate. This metabolite activates γδ T cells and triggers the release of proinflammatory cytokines such as interleukin‐6, tumor necrosis factor‐α, and interferon‐γ, resulting in APRs. The response is thought to be amplified by depletion of geranylgeranyl pyrophosphate in the mevalonate pathway [12]. This side effect is estimated to occur in 30%–40% of patients during the first infusion, commonly within the first 3 days [13]. In patients with AI, the associated cytokine surge may create sufficient physiological stress to precipitate an adrenal crisis. Rossini et al. [13] identified higher baseline counts of circulating γδ T cells, younger age, and receiving a first ZA infusion as predictors for APR. In the same study, vitamin D levels were not found to be a significant determinant of APR, whereas Crotti et al. reported that low vitamin D levels were associated with an increased risk [14]. In our reports, both patients were receiving their first ZA infusion, were vitamin D replete, and γδ T‐cell testing was unavailable. Previous case reports have shown similar observations, which are summarized in Table 3.

**Table 3:** Cases regarding zoledronic acid use in patients with adrenal insufficiency and the precautionary measures taken.

Case	Age	Sex	Etiology	Hydrocortisone dose (mg)	Bisphosphonate agent (mg)	Precautionary measures (mg)	Onset of crisis (h)
Basu et al. [7]	51	Female	Secondary AI	10 + 5	Zoledronic acid [5]	Paracetamol (650)	24
Kuo et al. [8]	70	Female	Primary AI	2.5	Zoledronic acid [5]	None	24
Smrecnik et al. [9]	80	Female	Primary AI	10 + 5 + 5	Zoledronic acid [5]	Paracetamol (500) and double the steroid evening dose	24
Case 1	60	Female	Secondary AI	10	Zoledronic acid [5]	Paracetamol (500)	24
Case 2	50	Female	Secondary AI	10 + 5	Zoledronic acid [5]	Paracetamol (500)	48

ZA may have a specific complication in inducing adrenal crises, as neither patient developed an adrenal crisis before its use, despite the usual flu, common sickness, and annual influenza vaccinations during their lives, which is beyond the physiological reaction of only APRs. Moreover, the occurrence of adrenal crises in patients with secondary AI is rare, with an incidence of 3.6 adrenal crises (95% confidence interval: 3.1–4.1) per 100 patient‐years among 319 patients with secondary AI [15]. This suggests that ZA influences cortisol metabolism in a manner distinct from that of APRs. However, further studies are needed to confirm these hypotheses.

Several precautionary measures have been proposed to mitigate the risk of APRs during ZA infusion. The use of antipyretics and dexamethasone for several days has been shown to reduce the risk of APRs [16–18]. The combination of dexamethasone and acetaminophen has a synergistic effect [19]. In our case and others [9–11], the use of acetaminophen did not seem to provide sufficient protection against the risk of APRs.

Statin therapy has received attention as a precautionary measure against APRs following ZA administration. Although in vitro experiments have shown promising results [20], several randomized trials have failed to demonstrate the clinical benefits [17, 21–23]. Our patients had dyslipidemia and were administered statins for a prolonged period prior to infusion. At the time of writing this report, there is an ongoing trial examining pravastatin, which is thought to have a stronger inhibitory effect owing to its weaker protein‐binding properties [24].

Bisphosphonates, including ZA, have historically been known to be the first line of treatment for osteoporosis. However, our cases suggest a potential risk of adrenal crisis with their use in patients with AI. While no trials have specifically examined the safety of osteoporosis therapies in this population, oral bisphosphonates carry a lower risk of APRs and, theoretically, seem to be a safer alternative [25, 26]. Denosumab, a monoclonal antibody, is not known to cause APRs and may represent another suitable option [27]. In both of our patients, denosumab was administered and did not exhibit any risk of APRs. The administration of ZA to patients with AI may pose a risk of adrenal crisis owing to its induction of APRs. Our findings suggest that individuals with AI may constitute a unique subgroup of patients with osteoporosis who require caution before ZA administration. Furthermore, these patients warrant extra care by following recommended stress‐dose steroid regimens, as outlined in current clinical practice guidelines. For patients on hydrocortisone doses below 40 mg daily, the total daily dose should be increased to 40 mg, administered in three divided doses for 2–5 days starting on the day of infusion [28]. The medication can still be safely given in a day‐case setting as long as patients are appropriately covered with stress‐dose steroids and given clear instructions on monitoring for symptoms and when to seek medical attention over the next 24–48 h. Finally, our report highlights the potential benefits of selecting alternative anti‐resorptive therapies that may have a safer profile in patients with AI.

## Ethics Statement

This case series report was approved by the Institutional Review Board (IRB) of King Fahad Medical City, Riyadh Second Health Cluster, prior to data collection and manuscript preparation (Approval Number 24‐214).

## Consent

Full informed consent for publication of this case report and accompanying clinical information was obtained from both patients. All reasonable efforts were made to ensure anonymization.

## Disclosure

A preliminary version of these case reports was presented as a poster at ENDO 2024 (The Endocrine Society Annual Meeting) and published as an abstract in the *Journal of the Endocrine Society* [29].

## Conflicts of Interest

The authors declare no conflicts of interest.

## Funding

This case series did not receive any specific grant from funding agencies in the public, commercial, or not‐for‐profit sectors. The authors conducted this work as part of their regular clinical duties and did not receive any additional compensation or resources for preparing this manuscript.

## Data Availability

Data sharing is not applicable to this article, as no datasets were generated or analyzed during the current study.
